# Investigation of *CTNNB1* gene mutations and expression in hepatocellular carcinoma and cirrhosis in association with hepatitis B virus infection

**DOI:** 10.1186/s13027-020-00297-5

**Published:** 2020-06-03

**Authors:** Davod Javanmard, Mohammad Najafi, Mohammad Reza Babaei, Mohammad Hadi Karbalaie Niya, Maryam Esghaei, Mahshid Panahi, Fahimeh Safarnezhad Tameshkel, Ahmad Tavakoli, Seyed Mohammad Jazayeri, Hadi Ghaffari, Angila Ataei-Pirkooh, Seyed Hamidreaz Monavari, Farah Bokharaei-Salim

**Affiliations:** 1 Department of Virology, Iran University of Medical Sciences, Tehran, Iran; 2 Department of Biochemistry, School of Medical Sciences, Iran University of Medical Sciences, Tehran, Iran; 3 Department of Interventional Radiology, Firouzgar Hospital, Iran University of Medical Sciences, Tehran, Iran; 4 Gastrointestinal and Liver Diseases Research Center, Iran University of Medical Sciences, Tehran, Iran; 5 Student Research Committee, Iran University of Medical Sciences, Tehran, IR Iran; 6 Department of Virology, Tehran University of Medical Science, Tehran, Iran; 7 Research Center for Clinical Virology, Tehran University of Medical Sciences, Tehran, Iran; 8 HIV Laboratory of National Center, Deputy of Health, Iran University of Medical Sciences, Tehran, Iran

**Keywords:** HBV, HCC, β-Catenin, *CTNNB1*, Mutation

## Abstract

Hepatitis B virus (HBV), along with Hepatitis C virus chronic infection, represents a major risk factor for hepatocellular carcinoma (HCC) development. However, molecular mechanisms involved in the development of HCC are not yet completely understood. Recent studies have indicated that mutations in *CTNNB1* gene encoding for β-catenin protein lead to aberrant activation of the Wnt/ β-catenin pathway. The mutations in turn activate several downstream genes, including *c-Myc*, promoting the neoplastic process. The present study evaluated the mutational profile of the *CTNNB1* gene and expression levels of *CTNNB1* and *c-Myc* genes in HBV-related HCC, as well as in cirrhotic and control tissues. Mutational analysis of the β-catenin gene and HBV genotyping were conducted by direct sequencing. Expression of β-catenin and *c-Myc* genes was assessed using real-time PCR. Among the HCC cases, 18.1% showed missense point mutation in exon 3 of *CTNNB1*, more frequently in codons 32, 33, 38 and 45. The frequency of mutation in the hotspots of exon 3 was significantly higher in non-viral HCCs (29.4%) rather than HBV-related cases (12.7%, *P* = 0.021). The expression of β-catenin and *c-Myc* genes was found upregulated in cirrhotic tissues in association with HBV infection. Mutations at both phosphorylation and neighboring sites were associated with increased activity of the Wnt pathway. The results demonstrated that mutated β-catenin caused activation of the Wnt pathway, but the rate of *CTNNB1* gene mutations was not related to HBV infection. HBV factors may deregulate the Wnt pathway by causing epigenetic alterations in the HBV-related HCC.

## Introduction

Hepatocellular carcinoma (HCC) is the most occurring malignancy of the liver and with 782,000 new cases annually and 600,000 deaths/year globally accounts for at least 75% of all liver cancer cases [1, 2]. Major risk factors are chronic infections with Hepatitis B Virus (HBV) and Hepatitis C Virus (HCV), alcohol, aflatoxin and fatty liver diseases; however, HBV is implicated as the most common etiological agent of HCC worldwide [2, 3]. The highest incidence of HCC is seen in Eastern Asian countries followed by the Asian-Pacific region, and these regions have high rates of HBV and HCV. Hepatic carcinogenesis is a multistep process characterized by multiple genetic alterations whose mechanisms are not fully understood [1, 2].

Genetic mutations and aberrant activation of signal transduction pathways play important roles in the development of HCC. It has been confirmed that several signaling pathways are aberrantly activated in HCC development [4]. Among these, the Wnt/β-catenin pathway seems to play a pivotal role in initiating and sustaining HCC development [5].

In the canonical Wnt pathway, β-catenin is the main effector normally sequestered by the destruction complex [6]. In the absence of Wnt ligands, β-catenin is phosphorylated by GSK3β, and subsequently ubiquitinated and degraded in the proteasome [7]. However, in the presence of ligands (Wnt proteins), Wnts interact with receptors (FZDs) and co-receptor molecules (LRP5/6), thereby disassembling the destruction complex [8]. This signal leads to cytoplasmic stabilization of β-catenin and then nuclear transport of β-catenin. In the nucleus, β-catenin triggers activation and transcription of downstream target genes through conjunction with TCF/LEF DNA binding proteins. Next, the consequence is stimulation of target genes involved in the cell proliferation and/or carcinogenesis with their proto-oncogenic effect. Wnt target genes include *c-Myc, cyclin D1, CTGF, WISP2* and *c-fus*, among which upregulation of *c-Myc* leads to proto-oncogenic effect in liver malignancies [8–10].

Mutations in the β-catenin gene are in association with the development of several human cancers, including HCC [11–13]. Alterations in phosphorylation targets in the N-terminal domain of β-catenin prevents the phosphorylation of β-catenin and subsequent proteasomal degradation. This leads to cytoplasmic accumulation of β-catenin, which in turn translocates to the nucleus and stimulates the transcription of Wnt target genes involved in apoptosis and proliferation [6].

Genetic alteration in liver malignancies shows diverse patterns in different geographical regions which is most likely linked to risk factors and mutations in human genes [12, 14]. For example, according to the COSMIC database *CTNNB1* mutation rate is higher in Europe and Americas than in Asia [12]. Several investigations have addressed the association of *CTNNB1* mutations in HCV-associated HCCs [12, 15, 16]. However, few studies with small sample sizes have assessed *CTNNB1* mutations in HBV-related HCCs. Moreover, no data was available regarding this pattern in the Middle East and Iran. Therefore, we analyzed the mutational pattern of *CTNNB1* gene in a series of HBV positive HCC specimens obtained from a cohort of Iranian patients.

## Material and methods

### Patients and samples

Fifty-four fresh frozen (FF) tissues, comprising cases of HCC, liver cirrhosis (LC) and normal histology tissues, were taken by fine needle biopsies from patients attending hospitals affiliated with Iran University of Medical Sciences. Moreover, formalin fixed paraffin embedded (FFPE) samples (*n* = 111) including HCC, LC and non-tumor tissues were also retrospectively analyzed. Overall, 165 samples were examined, including HBV positive HCC (*n* = 71), non-virus related HCC (*n* = 34), liver cirrhosis (*n* = 28) and tissues with normal histology (*n* = 32). Demographic and clinical information of the patients are briefly shown in Table 1.

**Table 1:** Demographic and clinical information of the studied population

Characteristics	HBV-HCC	Non-viral HCC	LC	Normal liver
Number	71	34	28	32
Male	55 (77.5)	24 (70.6)	15 (53.6)	20 (62.5)
Female	16 (22.5)	10 (29.4)	13 (46.4)	12 (37.5)
Age				
Mean	60.2 ± 12.4	59.2 ± 14.2	57.8 ± 13.6	50.5 ± 14.5
< 40	4 (5.6)	4 (11.8)	4 (14.3)	7 (21.9)
40–50	8 (11.3)	3 (8.8)	3 (10.7)	8 (25)
51–60	19 (26.8)	5 (14.7)	11 (39.3)	11 (34.4)
> 60	40 (56.3)	22 (64.7)	10 (35.7)	6 (18.8)
HBV+	71 (100)	0	12 (42.8)	11 (34.4)
Cirrhosis	20 (28.2)	7 (20.6)	28 (100)	–

Archived samples were collected from March 2013 and FF samples from May 2017 to April 2019. The FF and FFPE samples were histologically examined by an expert pathologist for confirmation of HCC, LC or normal histology. Informed consent was signed by each patient, and the project was approved by the ethical committee of research at IUMS under declaration of Helsinki (Ethic code: IR.IUMS.FMD.REC 1396.9321540004).

### DNA extraction

DNA was isolated using tissue DNA extraction kit (NucleoSpin® Tissue, Mini kit for DNA from cells and tissue, Macherey-Nagel Co, Germany) according to the kit instructions with the use of 1 mg of liver tissue. For each FFPE sample, 10 cuts with 15 μm thickness were first underwent for paraffin removal using xylene and ethanol, and then 1 mg of obtained tissue was forwarded in the above DNA isolation kit. The concentration and purity of isolated DNA were checked by BioPhotometer (NanoDrop 1000 Spectrophotometer, Thermo Fisher Scientific, United States) and running in agarose gel.

### RNA extraction and synthesis of cDNA

Isolation of total RNA from FF samples was performed using a tissue RNA extraction kit (NucleoSpin® total RNA, MN, Macherey-Nagel Co, Germany). For each FFPE samples, 10 cuts with 10 μm thickness were subjected for paraffin removal. We performed additional processing steps to improve RNA extraction from FFPE as described in the supplementary method. Then, the processed samples were used for RNA extraction with the above-mentioned kit. The purity and concentration of obtained RNA were checked by a BioPhotometer (The ratio of 260/280 was optimized between 1.7–2). The concentration and integrity of RNA were observed on agarose gel (Supplementary Figure 1). For gene expression assays, RNA samples were first treated with DNase I (Yekta Tajhiz Azma, YTA, Iran), and then DNase treated RNAs were applied to synthesize cDNA using random hexamer primers in the procedure of the First Strand cDNA Synthesis Kit (Fermentas, Thermo Fisher Scientific TR Limited, Waltham, Massachusetts, United States).

### Identification of mutations in *CTNNB1* gene

Several standard PCR assays were designed to identify β-catenin mutations. In all PCR assays of this study, necessary care was taken to avoid any carry over contamination. All the primers used in this study were synthesized and purchased from Metabion Company (Metabion international AG, Planegg/Steinkirchen, Germany, Table 2).

**Table 2:** The sequence of primers used for amplification of CTNNB1 gene, HBsAg coding region and gene expression analysis

Gene	Forward 5′- 3′	Revers 5′- 3′	PCR size	Ta_a_	Ref
β-catenin	CGTGGACAATGGCTACTCAA	CACTCAGAGAAGGAGCTGTGG	150	57	^b^
c-Myc	GGACGACGAGACCTTCATCAA	CCAGCTTCTCTGAGACGAGCTT	92	60	[10]
HMBS	CCCTGCCAGAGAAGAGTGTG	GTGTTGAGGTTTCCCCGAAT	109	57	^b^
GAPDH	CGACCACTTTGTCAAGCTCA	AGGGGTCTACATGGCAACTG	228	58	[17]
HBS1	GAGTCTAGACTCGTGGTGGACTTC	AAATKGCACTAGTAAACTGAGCCA	448	58	[18]
HBS2	CGTGGTGGACTTCTCTCA ATTTTC	GCCARGAGAAACGGRCTGAGGCCC	417	60	[18]
CTNNB1 ex3–5	TAGCTGATTTGATGGAGTTGG	CTCACGATGATGGGAAAGGT	994	57	^b^
CTNNB1 ex3, outer	TGCTTTTCTTGGCTGTCTTTC	CCTAAATGGTAAAAGTGACATTGC	500	55	^b^
CTNNB1 ex3, inner	TGCTAATACTGTTTCGTATTTATAGC	TTCTGACTTTCAGTAAGGCAATG	293	53	^b^

^a^Ta is the temperature of annealing for each primer set

^b^These primers were designed in current study

A single step PCR was designed to amplify exons 3 to 5 (Table 2). The thermal conditions were as follows: pre-heat at 95 °C for 10 min, then 45 cycles of 94 °C for 15 s, 57 °C for 15 s, and 72 °C for 1 min for amplification a 994 base pair product. Moreover, a nested PCR approach was designed for whole exon 3 of the *CTNNB1* gene (Table 2).

### Determination of the expression levels of β-catenin and *c-Myc*

To assess the active status of the Wnt pathway, the expression levels of β-catenin and *c-Myc* mRNAs were evaluated using the SYBR green based real-time PCR assay. Primers used in this section are given in Table 2. All the RT- PCR programs were performed on a Rotor Gene 3000 Real-Time PCR Machine using SYBR master 2X (2X SYBR® Green Real Time PCR Master Mix, Pars Tous Biotechnology, Mashhad, Iran). Samples were tested in duplicates and mean cycles of threshold (Ct) was used for further analyses. Values were normalized relative to the expression level of two internal housekeeping genes (GAPDH and HMBS) [17]. The 2^-ΔΔCt^ formula was used for calculation of relative gene expression. In this way, ΔCt means Ct of target gene –mean Ct of GAPDH and HMBS, and ΔΔCt is equal to tumor ΔCt- normal sample ΔCt [19].

### Genotyping of HBV

HBV infection of all samples was investigated using a conventional PCR test, as previously described [18]. Table 2 presents the sequence of primers used; the nested-PCR amplicon was a 417 bp segment corresponding to the HBsAg coding region subsequently subjected to direct sequencing for HBV genotyping. The obtained sequence data were aligned with the reference sequence of the accession number GQ183486 for construction of the phylogenetic tree using the MEGA software version 7.

### Statistical analysis

The data were categorized in the SPSS package and compared using chi-square, x^2^ test or Fisher’s exact tests. Values lower than 0.05 were considered as significant. Mann-Whitney and student t-test were used to compare quantitative characters. The Graph Pad Prism software (Version 6) was used for graphical analysis of gene expression data.

## Results

### Viral characteristics

All samples were investigated for HBV-DNA, and 56.9% were found to be positive for HBV; 71/105 (67.6%) among HCC cases, 12/28 (42.8%) in the cirrhosis group and 11/32 (34.4%) among liver samples with normal histology (Table 1). In the HCC group, the HBV S gene was partially sequenced and the nucleotide sequences subjected to phylogenetic analysis (Data are not shown) by using the MEGA software and also Geno2Pheno online tool (https://hbv.geno2pheno.org/). The results showed that all characterized samples belonged to the HBV subgenotype D1.

### Frequency of mutations in *CTNNB1* gene

Exon 3 and exons 3–5 were amplified using a pfu Taq-DNA polymerase (Yekta Tajhiz Azma, YTA, Iran) (Supplementary Fig. 2), and subjected to sequencing (Bioneer, South Korea). In exon 4, two point mutations (Q85H and D145E) were found only in non-viral HCC (Supplementary Fig. 3). In exon 5, there were two point mutations (Q203H and E209Q); no mutation was identified in both normal and LC group.

**Fig. 1:**
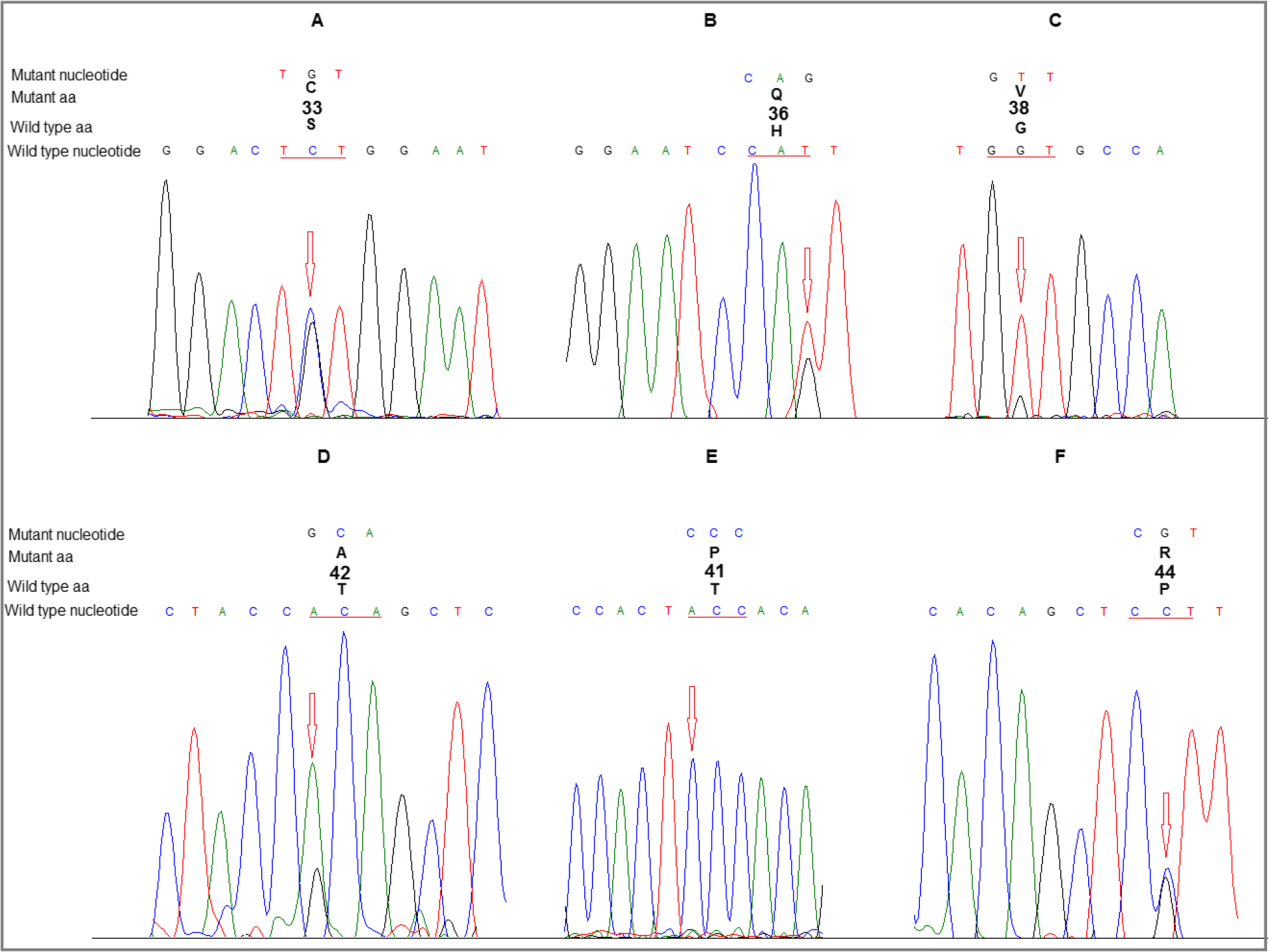
Examples of representative sequence chromatograms of the identified mutations in the hotspot region of β-catenin gene. A: S33C, B: H36Q, C: G38V, D: T42A, E: T41P, F: P44R

**Fig. 2:**
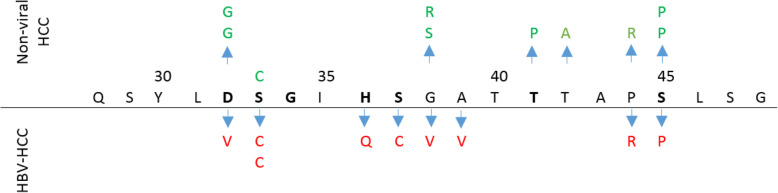
Illustration of the location of N- terminal amino acids of β-catenin along with the profile of mutations identified in HBV-HCC and non-viral HCC. Phosphorylation positions were marked with bold letters

Overall, 19/105 of HCC samples (18.1%) had at least one non-synonymous mutation in the hotspot region of β-catenin (Table 3, and Fig. 1). Known mutations found in exon 3 were: D32V/G, S33C, H36Q, S37C, G38V/S/R, A39V, T41P, T42A, P44R and S45P. The majority of mutations was observed in codons 32 (n: 3), 38 (n: 3) and 45 (n: 3), in which serine was the most affected amino acid (Fig. 2). No deletion or insertion was identified in the studied regions. However, there were some other unknown missense and nonsense mutations listed in the supplementary table. Among LC and normal tissues, there was no mutation in exon 3 of the β-catenin gene.

**Table 3:** CTNNB1 mutation in HCC cases according to demographic and pathological characteristics

Characteristics	CTNN mutation N (%)	P
Male	14 (17.7)	0.575
Female	5 (19.2)	
**Age**		
40 >	1 (12.5)	0.034^a^
40–50	1 (9.1)	
51–60	4 (16.6)	
> 60	13 (21)	
**Differentiation**		
Well	9 (28.1)	0.097^b^
Moderate	5 (16.7)	
Poorly	4 (15.4)	
Un-known	1 (5.9)	
**LC-HCC**		
Yes	4 (14.8)	0.423
No	15 (19.2)	

^a^The frequency of mutations was significantly increased toward aging, ^b^The rate of mutations in well differentiated tumors was higher than others

### Mutations of *CTNNB1* in association to HBV infection

The frequency of mutations in the hotspots of exon 3 was significantly higher in non-viral HCC rather than HBV-related ones (*P* = 0.021). Overall, 9/71 (12.7%) of HBV-HCCs, and 10/34 (29.4%) in non-viral HCCs had a mutation in the hotspot region of β-catenin. Furthermore, the pattern of mutations was also different between HBV-HCC and non-viral HCC (Fig. 2). A mutation in codon 33 (S33C) was the most prevalent mutation found in three HBV-HCC samples. Some mutations were observed more prevalently in HBV-HCCs; S33C (n: 3), H36Q (n: 1), S37C (n: 1) and A39V (n: 1); however, other mutations were seen in both groups (S33C, P44R and S45P). Tissues with non-viral HCC had a higher frequency of mutation in codon 32, 38 and 45 rather than HBV-related ones (5.8% vs 1.4% for each mutation). Moreover, the type of amino acid change was also different in these positions: G vs Y in 32 and R vs V in 38 (Fig. 2). The frequency of mutation in codon 44 was equal, and the type of change was also similar in tumors with both etiologies (P44R). Nevertheless, T41I and T42A mutations were identified only in non-viral tumors.

### Determination of β-catenin expression level

**Fig. 3:**
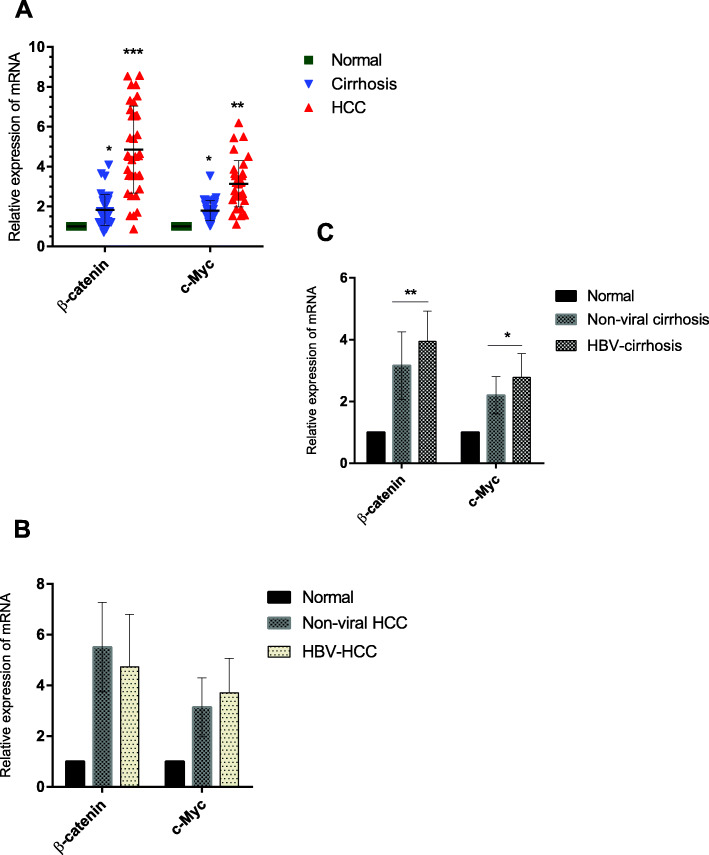
The results of real-time PCR for β-catenin and *c-Myc* were analyzed in normalization to the mean expression level of GAPDH and HMBS and in adjustment to the control group. The result showed that the expression of β-catenin and *c-Myc* was significantly upregulated among cases with LC and HCC (**a**). The expression among HBV-HCC and non-viral HCC was not statistically different (**b**). The expression of these genes showed elevated levels among HBV-LC (**c**). *: *p* < 0.05, **: *p* < 0.005, ***: *p* < 0.0005, ****: *p* < 0.00005

**Fig. 4:**
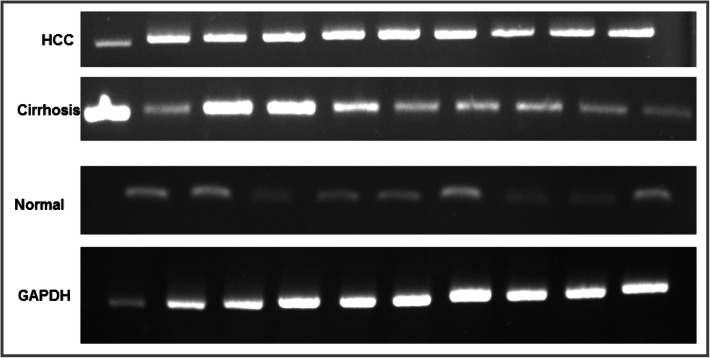
RT-PCR product of β-catenin was resolved in agarose gel, and the intensity of the bands were semi-quantified with GelCount™ software. Elevated expression of β-catenin in HCC and LC groups was observed in comparison to controls. All the lanes represent the samples tested

The results of real-time PCR showed that the expression level of β-catenin was significantly higher among the tumor samples than in the normal group (Fig. 3, Fig. 4). Elevated levels of β-catenin and *c-Myc* expression were observed also in cirrhotic samples (Fig. 3, Fig. 4). Higher levels of β-catenin and *c-Myc* were found in the HBV-LC group than non-viral-LC group; however, the expression level of *c-Myc* was not statistically different between HBV-HCC and non-viral HCC groups (Fig. 3).

### Determination of the expression level of β-catenin in association to mutations

Next, we aimed to determine whether the mutation phenotypes identified in HCC tumors were associated with the activation of the Wnt pathway. Considering that *c-Myc* is one of the target genes of this pathway [20], Wnt activation was assessed by analyzing *c-Myc* expression levels following normalization to the GAPDH gene.

**Fig. 5:**
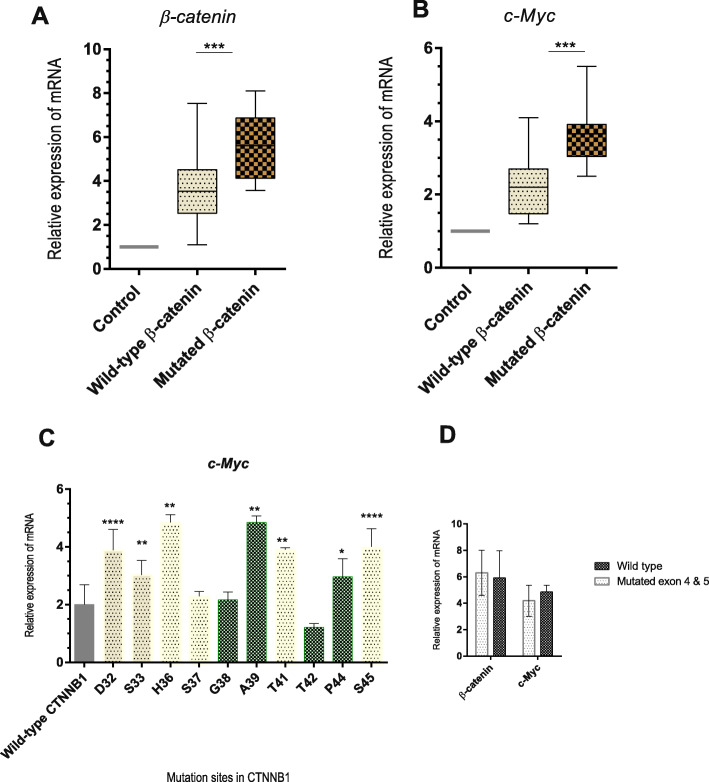
Real-time PCR for β-catenin and *c-Myc* assessed in normalization to the mean expression level of GAPDH and HMBS in adjustment to the control group. The result showed that the expression of β-catenin and *c-Myc* was significantly upregulated among cases with known hot spot mutations (**a** and **b**). Among samples with different mutations in exon 3 of the CTNNB1 gene, the level of *c-Myc* was evaluated in comparison to the samples with wild type CTNNB1 gene (**c**). Among samples with a mutation in exons 4–5 of the CTNNB1 gene, the level of *c-Myc* was somewhat similar to the wild type (**d**). *: *p* < 0.05, **: *p* < 0.005, ***: *p* < 0.0005, ****: *p* < 0.00005

Tumor samples harboring at least one missense mutation in the hotspot region of β-catenin revealed significant higher levels of *c-Myc* compared to wild type cases (Fig. 5). Among different mutation sites identified in this study, mutations at positions 32 and 45 were related to higher levels of *c-Myc*. Moreover, it appeared that mutation in both phosphorylation sites (32, 33, 36, 37, 41 and 45) as well neighboring sites within the hotspot region (39 and 44) could result in upregulation of *c-Myc* (Fig. 5). Among cases with mutation in exons 4 and 5, no significant difference was observed in term of gene expression (Fig. 5).

## Discussion

Molecular mechanisms involved in the development of HCC are not yet completely understood. This is partly owing to different etiologies of HCC as well as genetic heterogeneity of the patients. Overexpression and activation of β-catenin are now regarded as a frequent event in the development of HCC, which may be caused by mutations in the *CTNNB1* gene as well as some other epigenetic factors. Hence, we examined the mutation status in exons 3–5 of the *CTNNB1* gene and expression level of β-catenin gene in association with HBV infection.

Among HCC cases, we found two non-synonymous nucleotide changes in exon 4 and two in exon 5 of *CTNNB1* gene. These exons correspond to the N-terminus domain of β-catenin that can be a phosphorylation site for GSK3β or casein kinase-1, and/or a binding site for alpha-catenin in a complex with nuclear factors (TCF/ LEF) [21]. However, mutated cases in exons 4 and 5 did not show elevated levels of β-catenin compared to wild types that may be due to the low number of mutated samples. Although most previous studies were focused more on exon 3 among HCC patients, the present study found some unknown mutations in exons 4 and 5 for the first time. However, previous studies reported mutations in exons 4 and 5 in different cancers of unknown primary and some cancer cell lines [11, 22–24].

Totally, 18.1% of HCC cases were mutated in the β-catenin-exon 3; this is the first report from the Middle East and Iran. This rate was significantly higher in non-viral HCC rather than HBV-related HCC (29.4% vs 12.7%). Similar findings were reported in most of previous studies and the rate of mutation in HBV-HCC ranged from 8 to 19% [15, 25–32], and even higher as 44.1% [9]. Otherwise, this figure among HCV-HCC and/or non-viral HCC tumors was previously reported in high levels from 28 to 50% [33–36], being much higher than our study. Our results appear to be moderately lower than reports from France [37], Japan [16, 38], Italy [15] and Europe [28]; however, it should be noted that HCV is more prevalent in those regions. Additionally, HBV is the leading cause of HCC in Iran and the Middle East [39]. Accordingly, it appears that there is a geographical pattern of *CTNNB1* mutation in coordination with the epidemiologic profile of HBV/HCV as well as other risk factors such as aflatoxins and alcohol, being higher in more developed countries rather than developing and under-developed countries [12, 40]. Furthermore, some studies did not find any *CTNNB1* mutation in HBV-HCC tumors [13] or even among HCC cases with other etiologies among black Africans [41, 42]. The reason for these intense negative results is not yet fully understood. However, it is well known that genetic origin and heterogenecity affect the mutation rates. Studies have indicated that mutation pattern and frequencies can be variable according to heterogeneity in etiology, host genetic and geographical regions [15]. Moreover, among the known driver HCC genes, there is an obvious different geographical pattern of mutation for TP53 and RB1 genes that are related to ancestry genetic [40].

Moreover, we demonstrated that HBV-HCCs had a unique profile of *CTNNB1* mutation pattern; S33C, H36Q, S37C and A39V were observed mostly in HBV-HCC, some of them are also reported in previous studies [29, 43]. Of these, positions at 33 and 37 are the target phosphorylation sites for GSK3β [21], and H36 increases the activity of Wnt target genes [7].

Mutations of β-catenin have already been found to be associated with nuclear accumulation of β-catenin, leading to transactivation and overexpression of Wnt target genes including *c-Myc, cyclin D1, CTGF, and WISP2* [9, 20, 44]. Therefore, we assessed the activation status of the Wnt pathway according to the expression level of *c-Myc.* Mutations at positions 32 and 45 were mostly related to elevated levels of *c-Myc*. However, mutations in both phosphorylation sites and other sites within the hotspot region result in upregulation of *c-Myc*. This finding is in line with previous studies reporting an elevated level of *c-Myc* among mutated HCC tumors compared to normal or wild type samples [8, 9, 41]. Functional effects of these mutations were not previously investigated among clinical samples. Mutations in this region lead to interference in the phosphorylation of β-catenin by the destruction complex (Axin, APC, CK1, and GSK3-β) and prevent subsequent proteasomal degradation of β-catenin, resulting in cytoplasmic accumulation and translocation of β-catenin to the nucleus and activation of the pathway by contributing with Tcf/Lef transcription factors [20].

Mutations of β-catenin lead to steady activation of the Wnt pathway, which may occur early in some tumors [21]. Moreover, higher levels of β-catenin may lead to the release of this protein from the Axin-APC scaffold complex [45] and translocation of free molecules to the nucleus. Thus, elevated levels of β-catenin may lead to nuclear accumulation of β-catenin and activation of the pathway. The current study results showed the overexpression of β-catenin among tumor samples as well as *CTNNB1* mutated cases, being accompanied by overexpression of *c-Myc*. Some previous studies also demonstrated transcriptional dysregulation and overexpression of *CTNNB1* [9, 46]. This suggests that deregulation of the Wnt pathway begins at the transcription level, as well as previous findings that β-catenin expression is in accordance with the protein and mRNA level [9].

There was an elevated levels of β-catenin and *c-Myc* expression in LC group that was associated to HBV infection. This finding was previously described with decreased level of serum β-catenin from chronic hepatitis B, HBV-LC to HBV-HCC [47]. However, no mutation was found in *CTNNB1* among the LC group. This finding suggest that β-catenin mutations and other liver injuries such as cirrhosis are not necessarily cooperative risk factors of HCC, and so they may independently contribute to the development of HCC [48].

The most important limitation of this study was the lack of functional tests to assess the effect of viral factors on the Wnt pathway, as well as in-vivo and in-vitro based experiments for exact elucidation of HBV-related up- or downregulation in gene expression.

In conclusion, a significant number of β-catenin mutations were identified in this study that were related to the overexpression of β-catenin. Overexpression of this gene was correlated with higher expression of *c-Myc* as one of Wnt pathway target genes. Overexpression of β-catenin and *c-Myc* in LC appeared to be an earlier event before development of HBV-HCC. HBV infection was not involved in the occurrence of mutations in the β-catenin gene; therefore, dysregulation of the Wnt pathway may be mediated by the effect of viral factors on other role players in the pathway. We found some unknown mutations in exons 4 and 5 of β-catenin that need further investigations in larger sample sizes to evaluate of the exact effect.

## Supplementary information

**Additional file 1: Figure S1.** The electrophoresis of RNAs on agarose gel. **Figure S2.** PCR products of β-catenin exons 3–5 on agarose gel. **Figure S3.** Unknown point mutations observed in exons 3–5 in the β-catenin among HCC samples. **Table S1.** List of mutations observed in this study.

## Data Availability

Data and complementary material are available and was uploaded in supplementary file.

## Abbreviations

HCC: hepatocellular carcinoma
HBV: hepatitis B virus
LC: liver cirrhosis
Wnt: Wingless-related integration site (Wingless family protiens)
c-Myc: cell- Myelocytomatosis
GSK3β: Glycogen synthase kinase 3 beta
FZD: frizzled class receptors
LRP: Low-density lipoprotein receptor-related protein
Axin: Protein Phosphatase, Component of the beta-catenin destruction complex required for regulating CTNNB1 levels
APC: Adenomatous polyposis coli
TP53: Tumor protein p53
CTNNB1: Catenin (Cadherin-Associated Protein), Beta 1, 88 kDa
GAPDH: Glyceraldehyde-3-Phosphate Dehydrogenase
HMBS: Hydroxymethylbilane Synthase
RB1: Retinoblastoma-associated protein
CTGF: CCN2, connective tissue growth factor
WISP: (CCN5) WNT1 inducible signaling pathway protein subfamily
CK1: Casein kinase 1 family
