# Supplementary material for: Investigation of CTNNB1 gene mutations and expression in hepatocellular carcinoma and cirrhosis in association with hepatitis B virus infection
Source: Infect Agent Cancer. 2020 Jun 3;15:37. doi: 10.1186/s13027-020-00297-5 (PMC7268324; doi:10.1186/s13027-020-00297-5)
Supplement: Supplementary file 1 — Additional file 1: Figure S1. The electrophoresis of RNAs on agarose gel. Figure S2. PCR products of β-catenin exons 3–5 on agarose gel. Figure S3. Unknown point mutations observed in exons 3–5 in the β-catenin among HCC samples. Table S1. List of mutations observed in this study. [file 13027_2020_297_MOESM1_ESM.pdf]

Supplementary material

https://doi.org/10.1186/s13027-020-00297-5

Investigation of *CTNNB1* gene mutations and expression in hepatocellular carcinoma and cirrhosis in association with hepatitis B virus infection

Davod Javanmard, Mohammad Najafi, Mohammad Reza Babaei, Mohammad Hadi Karbalaie Niya, Maryam Esghaei, Mahshid Panahi, Fahimeh Safarnezhad Tameshkel, Ahmad Tavakoli, Seyed Mohammad Jazayeri, Hadi Ghaffari, Angila Ataei-Pirkooh, Seyed Hamidreaz Monavari *, Farah Bokharaei-Salim*

Contents:

1- RNA extraction from FFPE samples
2- Figure 1
3- Figure 2
4- Figure 3
5- Table 1
6- References

1- RNA extraction from FFPE samples

Background: Tissue autolysis is one of most significant element affecting the integrity of isolated RNA from FFPE samples, by the effect of nucleases that occurs between the times of sampling to fixation. Additionally, it was shown that thickness of the specimens has direct association with increasing the nucleases effect due to reduced rate of formalin penetration and inactivation of enzymatic activity in more thick sections [1]. In this term, since most of liver biopsies are taken through needle biopsy and the sections are fine thin, the obtained RNA yield from liver FFPE samples is more reliable for gene expression profiling [2, 3]. Moreover, formaldehyde is responsible for cross linking of RNA to another proteins or nucleic acids, which reduces the yield of isolated RNA. In this regard, we tried to reverse the effects of formalin, at least in some, by buffer treatment and washing as indicated previously [4, 5]. Extensive heating can be used for removal of formalin adduct from the nucleic acids, but this method cause extensive degradation of RNA due to overheating [6]. So, we tried to optimize RNA extraction and subsequent RT-PCR assays by the benefit of PBS treatment-washing, as well as using proteinase K and soft grinding the tissue before extraction of RNA. Moreover, the poly-A tail of RNA are most potent component to degrade upon FFPE processing, so cDNA synthesis by random hexamer primers have shown to improve the quality of mRNA expression profiling from the FFPE samples [7], as we performed in this research.

Protocol: For each FFPE sample, 10 cuts with 10 μm thickness were subjected to paraffin removal and additional processing before using the kit. The cuts were put in a 1.5 μL tube, then 1 mL xylene was added. The tube was vortexed vigorously and incubated at 50º C for 1 hour. Thereafter tubes were centrifuged at 13000^{rpm} for 3 min. The supernatant was removed with care and the step was repeated by adding 1 mL xylene. The pellet was washed with 100% ethanol 3 times, and finally air-dried. Dried tissues were immersed in PBS buffer and incubated at 4º C with occasional vortex. After 1-2 hours, the tubes were centrifuged at 13000^{rpm} for 1 min and the PBS was replaced; this process was repeated between 3-5 times. The tubes were centrifuged again and after removal of the PBS, the pellet was partially dried. Then the samples were subjected to tissue RNA extraction kit (NucleoSpin® total RNA, MN, Macherey-Nagel GmbH & Co. KG).

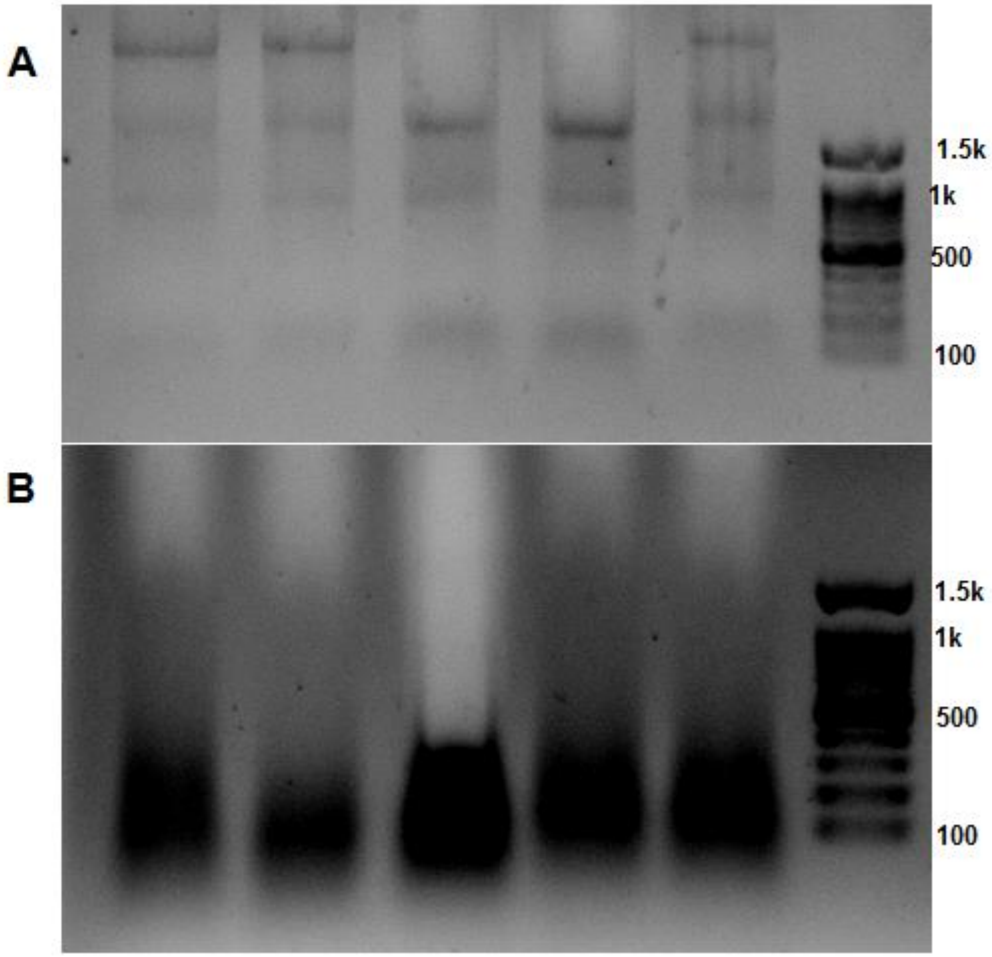

2- S. Figure 1. Ten µL of extracted RNA was run on 1% agarose gel with TBE buffer. A: FF-RNA, and B: FFPE-RNA

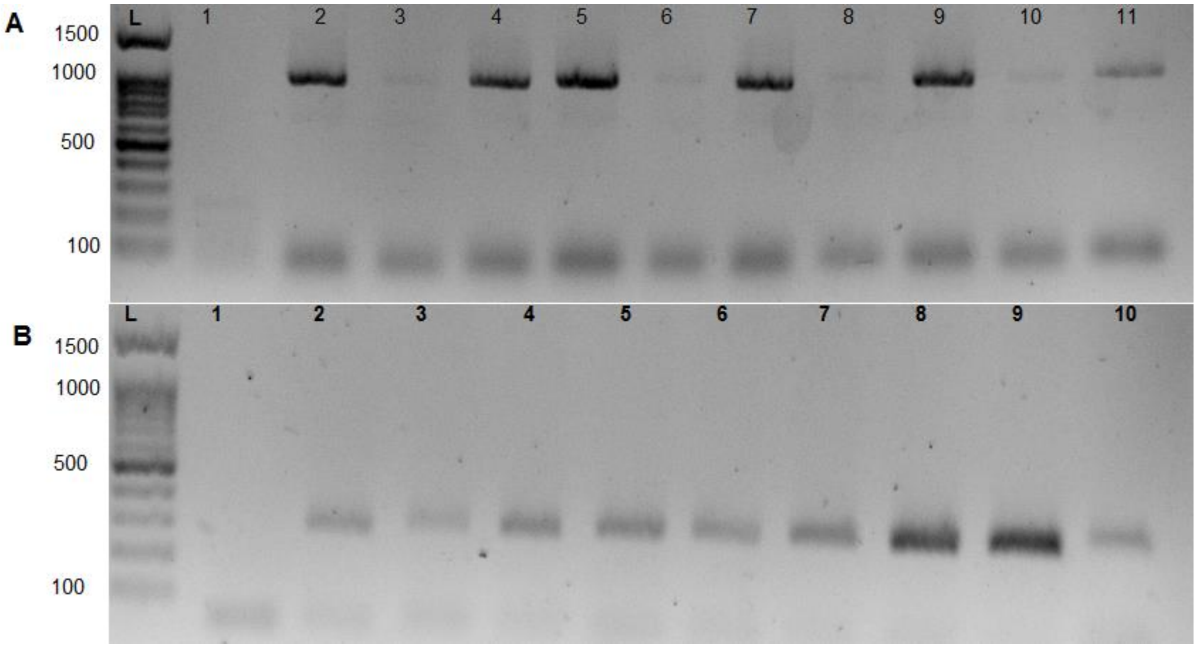

3- Figure 2. PCR products of β-catenin exon 3-5 (A) and exon 3 (B) were run on agarose gel. L: ladder, Lane 1: negative control, Lane 2: positive control, and the rest are samples.

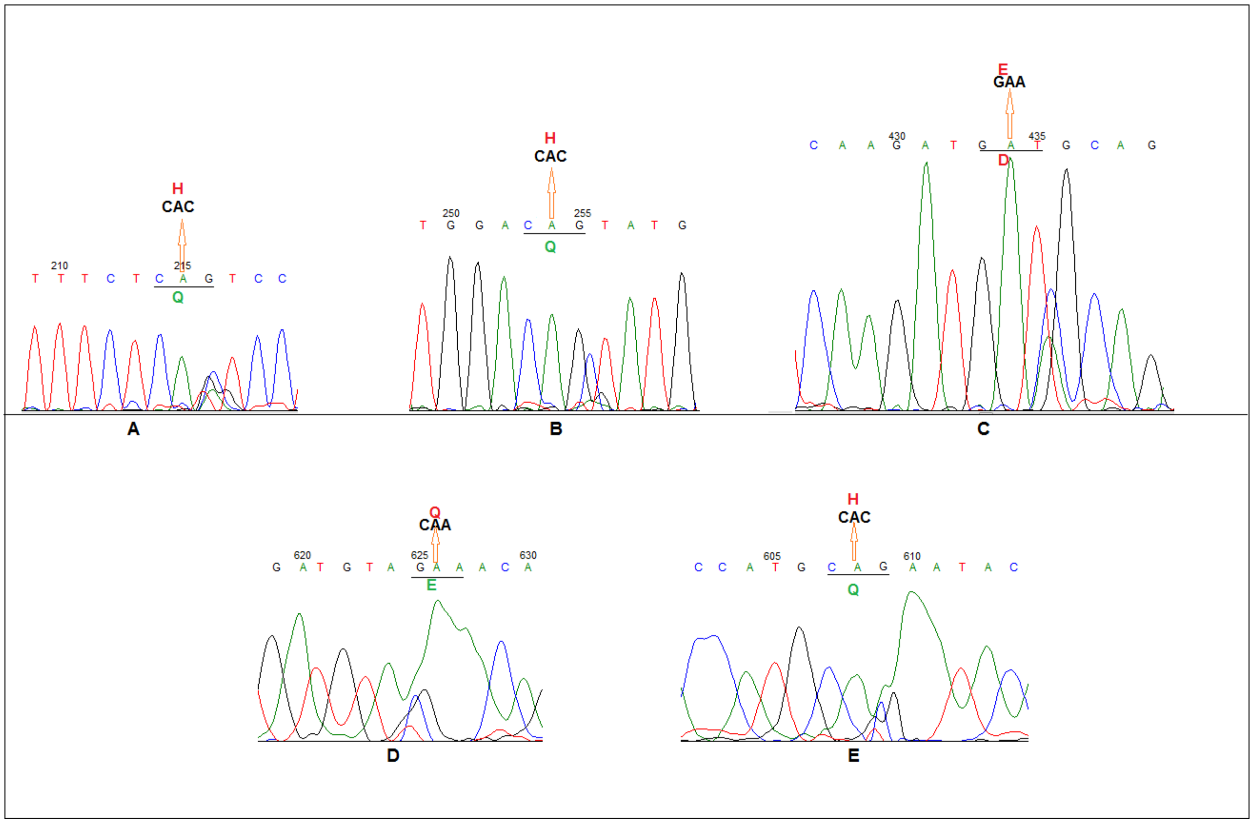

4- Figure 3. Unknown point mutations observed in exons 3-5 in the β-catenin among HCC samples. **A:** Q72H is in the exon 3, **B:** Q85H and **C:** D145E are within exon 4, **D:** E209Q and **E:** Q203H are in the exon 5. Green letters below the codon specify the wild type amino acid, red one above the mutant code represents the mutated amino acid. The number ordering of nucleotides begin from the start codon of β-catenin gene (NG_013302).

5- Table 1. list of mutations observed in this study

Sample code	Codon	Wild type codon	Mutant codon	Exon	Function
P31hc	43	GCT	GCC	3	Nonsense
P18hc	29	TCT	TCC	3	Nonsense
39f.hp	48	GGT	GGG	3	Nonsense
P181h	D32G/V	GAC	GGC/GTC	3	interaction of β-catenin with Fbw1
P98h	S33C	TCT	TGT	3	phosphorylation sites for GSK-3β
125f	H36G	CAT	CAG	3	hot-spot
212f	S37C	TCT	TGT	3	phosphorylation sites for GSK-3β
83f.bh	G38V	GGT	GTT	3	hot-spot
P8.hc	G38S/R	GGT	AGT/CGT	3	hot-spot
F45.p1	A39V	GCC	GTC	3	hot-spot
P158	T41I	ACC	ATC	3	phosphorylation sites for GSK-3β
P66.h	T42A	ACA	GCA	3	hot-spot
P204.h	P44R	CCT	CGT	3	hot-spot
Pf32	S45P	TCT	CCT	3	phosphorylation site for casein kinase-1
Pf128	Q72H	CAG	CAC	3	New mutation
F1.hp		GGTGAAGAAA	GGTGAAAAAA	Intron	New mutation
F25.hp	Q85H	CAG	CAC	4	New mutation
P51f	D145E	GAT	GAA	4	New mutation
Pf43H		GGAGTAGTT	GGACTAGTT	Intron	New mutation

F21	Q203H	CAG	CAC	5	New mutation
F15	E209Q	GAA	CAA	5	New mutation

6- References

1. Von Ahlfen, S., et al., *Determinants of RNA quality from FFPE samples.* PloS one, 2007. **2**(12): p. e1261.
2. Rong, M., G. Chen, and Y. Dang, *Increased miR-221 expression in hepatocellular carcinoma tissues and its role in enhancing cell growth and inhibiting apoptosis in vitro.* BMC cancer, 2013. **13**(1): p. 21.
3. Pan, L., et al., *Decreased expression and clinical significance of miR-148a in hepatocellular carcinoma tissues.* European Journal of Medical Research, 2014. **19**(1): p. 68.
4. Evers, D.L., et al., *The effect of formaldehyde fixation on RNA: optimization of formaldehyde adduct removal.* The Journal of molecular diagnostics : JMD, 2011. **13**(3): p. 282-288.
5. Joshi, B.D., et al., *An effective method for extraction and polymerase chain reaction (PCR) amplification of DNA from formalin preserved tissue samples of snow leopard.* African Journal of Biotechnology, 2013. **12**(22).
6. Karmakar, S., et al., *Organocatalytic removal of formaldehyde adducts from RNA and DNA bases.* Nature chemistry, 2015. **7**(9): p. 752-758.
7. Mittempergher, L., et al., *Gene expression profiles from formalin fixed paraffin embedded breast cancer tissue are largely comparable to fresh frozen matched tissue.* PloS one, 2011. **6**(2): p. e17163.
